# Microdosing with psilocybin mushrooms: a double-blind placebo-controlled study

**DOI:** 10.1038/s41398-022-02039-0

**Published:** 2022-08-02

**Authors:** Federico Cavanna, Stephanie Muller, Laura Alethia de la Fuente, Federico Zamberlan, Matías Palmucci, Lucie Janeckova, Martin Kuchar, Carla Pallavicini, Enzo Tagliazucchi

**Affiliations:** 1 Departamento de Física, Universidad de Buenos Aires and Instituto de Física de Buenos Aires (IFIBA – CONICET), Pabellón I, Ciudad Universitaria (1428), CABA, Buenos Aires, Argentina; 2 Fundación para la Lucha contra las Enfermedades Neurológicas de la Infancia (FLENI), Montañeses 2325, C1428 CABA, Buenos Aires, Argentina; 3 Institute of Cognitive and Translational Neuroscience, INECO Foundation, Favaloro University, Buenos Aires, Argentina; 4 Department of Cognitive Science and Artificial Intelligence, Tilburg University, Tilburg, the Netherlands; 5 Forensic Laboratory of Biologically Active Substances, Department of Chemistry of Natural Compounds, University of Chemistry and Technology Prague, Prague, Czech Republic; 6 Department of Experimental Neurobiology, National Institute of Mental Health, Klecany, Czech Republic; 7 Latin American Brain Health Institute (BrainLat), Universidad Adolfo Ibañez, Santiago de Chile, Chile

**Keywords:** Human behaviour, Physiology

## Abstract

The use of low sub-perceptual doses of psychedelics (“microdosing”) has gained popularity in recent years. Although anecdotal reports claim multiple benefits associated with this practice, the lack of placebo-controlled studies severely limits our knowledge of microdosing and its effects. Moreover, research conducted in standard laboratory settings could fail to capture the motivation of individuals engaged or planning to engage in microdosing protocols, thus underestimating the likelihood of positive effects on creativity and cognitive function. We recruited 34 individuals starting to microdose with psilocybin mushrooms (*Psilocybe cubensis*), one of the materials most frequently used for this purpose. Following a double-blind placebo-controlled experimental design, we investigated the acute and short-term effects of 0.5 g of dried mushrooms on subjective experience, behavior, creativity (divergent and convergent thinking), perception, cognition, and brain activity. The reported acute effects were significantly more intense for the active dose compared to the placebo, but only for participants who correctly identified their experimental condition. These changes were accompanied by reduced EEG power in the theta band, together with preserved levels of Lempel-Ziv broadband signal complexity. For all other measurements there was no effect of microdosing except for few small changes towards cognitive impairment. According to our findings, low doses of psilocybin mushrooms can result in noticeable subjective effects and altered EEG rhythms, but without evidence to support enhanced well-being, creativity and cognitive function. We conclude that expectation underlies at least some of the anecdotal benefits attributed to microdosing with psilocybin mushrooms.

## Introduction

Over the last decade, the use of relatively small doses of psychedelics to enhance mental function has attracted a significant amount of interest from the general public [1–3] and the scientific community [4]. The 2021 Global Drug Survey reported that 22% of those who used the most popular psychedelics in the last 12 months did so in the context of this practice (frequently known as “microdosing”), with *≈*4% percentage in the total sample, and up to 17% according to an independent study [5, 6]. Microdosing is frequently undertaken to improve mood, cognitive function and mental concentration, as well as to enhance creativity and problem-solving skills [7–9], yet the effects of low doses of psilocybin or other serotonergic psychedelics have not been extensively investigated to date. However, the extrapolation of some of the effects usually found for higher doses suggests that low amounts of psychedelics could modify brain oscillatory activity, perception, cognitive functions and mood [10, 11], in turn, these effects could be variable among individuals, depending on traits such as suggestibility [12, 13] and absorption [14]. Moreover, research suggests that full doses serotonergic psychedelics are capable of producing lasting positive changes in behavior, personality and mental health [10, 11, 15, 16]. Furthermore, there is anecdotal evidence supporting that microdosing psychedelics can increase creativity and problem-solving abilities, as well as promote cognitive flexibility and positively affect empathy and reduce levels of mind wandering [8, 17–19]. Some individuals microdose to self-medicate for cluster headaches, depression and anxiety, among other conditions [20–22]. Indeed, it has been proposed that microdosing with psychedelics could have therapeutic value for the treatment of mental health disorders [23]. The use of low doses of psychedelics constitutes an attractive therapeutic model, since it could circumvent the potential issues associated with altered consciousness and challenging experiences elicited by higher doses [24].

Owing to its origin as an underground practice, microdosing lacks standardized procedures that are accepted and replicated by the community [25]. Different serotonergic psychedelics are used for this purpose, such as lysergic acid diethylamide (LSD), dimethyltryptamine (DMT) ingested with a monoamine oxidase inhibitor (MAOI)—as in the concoction known as “ayahuasca”—and psilocybin, the active compound of several mushrooms in the *Psilocybe* genus. The most frequently used compounds are LSD and psilocybin, the latter in the form of dried psychoactive mushrooms [7–9, 26]. There is considerable variability in dose and dosing schedules [25]. Perhaps the most popular dosing schedule was proposed by James Fadiman, consisting of one dosing day followed by two days without dosing [17]. Dosing periods are also highly variable, ranging between 1 week to several years [25]. In the case of psilocybin mushrooms, microdoses are within the range of 0.1 g to 0.5 g of dried mushroom material [18], with 0.1 g considered roughly equivalent to *≈*4.6 µg of LSD [26].

The efficacy of microdosing to enhance mood, creativity and cognition and to reduce anxiety and depression is supported by anecdotal accounts [17] and, more recently, by online surveys, observational, and open-label studies [7–9, 18–20, 27–29]. Unfortunately, these studies lack adequate controls and are based on self-selected samples, rendering them vulnerable to confirmatory bias. It is important to note that expectations (which are generally positive in the context of recent scientific studies) play an important role in the perceived effects of microdosing with psychedelics, both for researchers and participants [8, 30–32]. When restricted to studies that follow double-blind and placebo-controlled experimental designs, considerably less evidence supports the positive effects of microdosing. Indeed, low doses of LSD can have effects that are different (or even opposite) to those expected by individuals who microdose [33–36]. Nevertheless, other reports have documented positive and dose-dependent enhancements in mood, emotional cognition and aesthetic perception, as well as significant improvements in emotional state, anxiety, energy and creativity, among other relevant variables [26, 35, 37, 38]. Importantly, some of these results could be explained by unblinding of the experimental condition, i.e., by subjects correctly distinguishing the placebo from the active dose [26, 38].

In spite of several recent studies addressing the effects of microdosing on mental health, mood, creativity and cognition, the physiological and neurobiological levels have been less investigated. In terms of brain activity, a study using functional magnetic resonance imaging (fMRI) showed that a very low dose of LSD sufficed to alter the functional connectivity between the amygdala and several cortical regions; moreover, some of these changes were correlated with self-reported assessments of positive mood [37]. Electroencephalography (EEG) is capable of robustly identifying the acute effects of different psychedelics, which result in broadband increases in signal entropy and band-specific changes in spectral power [39–48]. Despite these promising results, to date only one study applied EEG to investigate the effects of low doses of LSD, finding dose-dependent reductions in broadband oscillatory power during resting state with eyes open and closed, as well as modulation of event-related potentials (ERPs) in a visual oddball paradigm [49].

Here, we investigated the effects of low doses of *Psilocybe cubensis* on behavior, creativity (divergent and convergent thinking), perception, cognition and the underlying brain activity (measured with EEG), with emphasis on controlling for expectation without introducing an artificial motivation for microdosing. We recruited individuals who were planning to start a microdosing protocol with their own mushroom material —regardless of their previous experience with microdosing—and who willingly adapted their schedule and dose to meet the standardized conditions of our research protocol. The experimental condition (gel capsule with either 0.5 g of dried *Psylocybe cubensis* or the same weight of inactive placebo) was unknown to both participants and experimenters, and was only revealed after conclusion of data collection and analysis.

## Materials and methods

The supplementary methods contain further details on recruitment, inclusion and exclusion criteria, blinding procedure, experimental setting, chemical analysis of the samples, questionnaires and tasks, as well as the motivation for their inclusion in this study.

### Participants

A total of 34 participants (11 females; 31.26 ± 4.41 years; 74 ± 17 kg [mean ± STD]) were recruited by word-of-mouth, social media advertising, and visits to workshops on psilocybin mushrooms and microdosing between December 2019 and August 2020. Participants reported 11 ± 14.9 previous experiences with serotonergic psychedelics, of which 1.5 ± 2.3 were considered “challenging”. Only 6 of them reported significant previous experience with microdosing. All participants were fluent in Spanish, had normal or corrected-to-normal vision, and successfully completed all instances of the experiment.

This study was conducted in accordance with the Helsinki declaration and approved by the Committee for Research Ethics at the Universidad Abierta Interamericana (Buenos Aires, Argentina), protocol number 0-1054. Written informed consent was obtained from all the participants. The experiments entailed no deception and participants were fully informed about the purpose of the study. After the study ended, the order of the conditions was unblinded to the participants. The subjects did not receive financial compensation for their participation. This study was registered at ClinicalTrials.gov before it started (NCT05160220).

### Experimental design

**Fig. 1:**
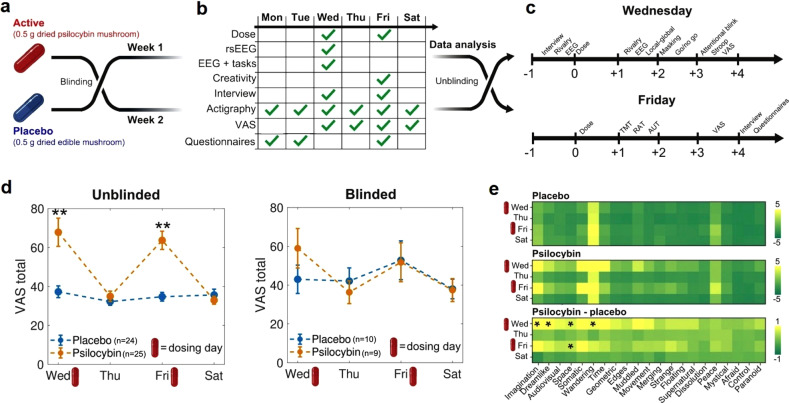
Experimental design and acute effects. **a** Neither the subjects nor the investigators knew the content of the capsules (active dose or placebo) until the last steps of the data analysis stage. Each condition (active dose or placebo) corresponded to 1 week of the experiment, separated by 1 week. **b** Measurements conducted during each day of the week. **c** Timelines for the measurements performed during dosing days (Fridays and Wednesdays). **d** VAS total score (mean *±* SEM) per condition, from Wednesday (first dosing day of the week) to Saturday (last day of the experiment), obtained from the “unblinded” (left) and “blinded” (right) subsets of the data. **p < 0.05, Bonferroni corrected (*n* = 4). **e** VAS scor**e**s per item, day of the experiment and experimental condition. The bottom matrix contains the difference between the active dose and the placebo. *p < 0.05, uncorrected for multiple comparisons.

This study followed a randomized, double-blind placebo-controlled, within-subjects design, outlined in Fig. 1. For each participant, the experiment was divided into 2 weeks (separated by 1 week without measurements), one corresponding to the active dose (0.5 g of ground and homogenized dried mushrooms in a gel capsule) and the other to the placebo (same weight and preparation, but using an edible mushroom). This dose is representative of the upper range used for microdosing [8, 18, 26, 38]. The order of these two conditions was randomized by a third party, who ensured that the identity of the capsules was unknown both to the participants and the researchers. This procedure is similar to the one implemented in a recent publication from the Center for Psychedelic Research, Imperial College, London (https://selfblinding-microdose.org/) [26], except that blinding was not performed by the participants themselves.

### Acute effects

Twenty-one items were adapted from Carhart-Harris et al. [44]. and presented in the form of VAS to determine the intensity of the acute effects experienced by subjects. The complete list of items can be found in the supplementary methods.

### Questionnaires and tasks

Participants completed self-reported scales aimed to assess psychological traits 2 days before the first dosing day of each condition. Afterwards, they performed different tasks and activities on Wednesdays and Fridays, and completed a battery of scales on Fridays. Table 1 summarizes all the tasks and measurements included in this study, and indicates the time points when they were obtained.

**Table 1:** Measures collected for each condition, including the domain they target, acronyms, and when they were obtained during the experiment (baseline, first dosing day, second dosing day, or daily).

Domain	Measure	Acronym	Mon	Tue	Wed	Thu	Fri	Sat
Acute subjective effects	Visual Analog Scale	VAS			**X**	**X**	**X**	**X**
Personality	Big Five Inventory	BFI	**X**					
Trait anxiety	State Trait Anxiety Inventory	STAI-T	**X**					
Suggestibility	Short Suggestibility Scale	SSS		**X**				
State anxiety	State Trait Anxiety Inventory	STAI-S					**X**	
State affect	Positive and Negative Affect Scale	PANAS					**X**	
Stress	Perceived Stress Scale	PSS					**X**	
Absorption	Tellegen Absorption Scale	TAS					**X**	
Well-being	Well-being Scale	BIEPS					**X**	
Mind wandering	Mind Wandering Questionnaire	MWQ					**X**	
Flow	Flow State Scale	FSS					**X**	
Creativity	Creative Personality Scale	CPS					**X**	
Empathy	Cognitive-Affective Empathy Test	TECA					**X**	
Cognitive flexibility	Cognitive Flexibility Scale	CFS					**X**	
Convergent thinking	Remote Associates Test	RAT					**X**	
Divergent thinking	Alternative Uses Task	AUT					**X**	
Divergent thinking	Wallach–Kogan Test	WK					**X**	
Perception	Binocular Rivalry	BR			**X**			
Conscious perception	Backward Masking	BM			**X**			
Attention, coordination	Trail Making Test	TMT					**X**	
Inhibition	Go / No Go	GNG			**X**			
Attention	Attentional Blink	AB			**X**			
Inhibition	Stroop Test	ST			**X**			
Brain activity	EEG with Eyes Open	EO			**X**			
Brain activity	EEG with Eyes Closed	EC			**X**			
Attention	Local-Global + EEG	LG			**X**			
Physical activity	Fitbit Charge 4 wristband	ACT	**X**	**X**	**X**	**X**	**X**	**X**

We assessed the following questionnaires (all in Spanish language): Big Five Inventory (BFI), State-Trait Anxiety Inventory (STAI-T / STAI-S), Short Suggestibility Scale (SSS), Positive and Negative Affect Schedule (PANAS), Mind Wandering Scale (MWQ*)*, Perceived Stress Scale (PSS), Tellegen Absorption Scale (TAS), Psychological Well-being Scale (BIEPS), Flow State Scale (FSS) Creative Personality Scale (CPS), Cognitive-Affective Empathy Test (TECA), and Cognitive Flexibility Scale (CFS).

The Spanish versions of creativity tests (also detailed in Table 1) included in our study were the Remotes Associates Test (RAT; convergent thinking), the Alternative Uses Task (AUT; divergent thinking), and the Wallach–Kogan Test (WK; divergent thinking).

We asked participants about their expectation of change in the following domains: positive emotion, negative emotion, anxiety, attention, absorption, creativity, perception, problem solving abilities, empathy, memory, energy level, sleep, sociability, spirituality, openness to new experiences, connectedness and use of psychoactive substances. The analysis of this data will be reported in a future publication.

Finally, the following computer-based tasks were implemented and used to determine the impact of psilocybin on perception and cognition: binocular rivalry (visual perception), backward masking (conscious visual perception), trial making test (attention and coordination), Go / No Go (inhibitory control), attentional blink (attention), and the Stroop test (inhibition). These tasks are described with detail in the supplementary methods, and are outlined in the upper panels of Fig. 3.

### Resting state EEG recording, preprocessing and analysis

EEG was recorded with a 24-channel research-grade mobile system (mBrainTrain LLC, Belgrade, Serbia; http://www.mbraintrain.com/) attached to an elastic electrode cap (EASYCAP GmbH, Inning, Germany; www.easycap.de). Twenty-four Ag/AgCl electrodes were positioned at standard 10–20 locations. Reference and ground electrodes were placed at FCz and AFz sites. The wireless EEG DC amplifier (weight = 60 g; size = 82 × 51 × 12 mm; resolution = 24 bit; sampling rate = 500 Hz, 0–250 Hz pass-band) was attached to the back of the electrode cap (between electrodes O1 and O2). EEG activity was acquired with eyes open and closed (5 min each) and during an auditory Local-Global paradigm.

EEG data was preprocessed using EEGLAB (https://sccn.ucsd.edu/eeglab/index.php) [50]. First, time series were bandpass-filtered (1–90 Hz) and notch-filtered (47.5–52.5 Hz). Channels with artifacts were detected using EEGLAB automated methods, with rejection criteria based on kurtosis (threshold = 5), probability (threshold = 5) and the rejection of channels with ±2.5 standard deviations from the mean in any parameter (rejected channels for psilocybin with eyes open: 2 *±* 1; for psilocybin with eyes closed: 1.5 *±* 0.9; for placebo with eyes open: 2 *±* 1; for placebo with eyes closed: 1.5 *±* 0.9 [mean *±* STD]). Channels were manually inspected before rejection and then interpolated using data from the surrounding channels. Next, time series were divided into 2 s epochs. Epochs to be rejected were flagged automatically and visually inspected before rejection. Infomax independent component analysis (ICA) was applied to data from each individual participant for the identification and removal of remaining recording artifacts.

The logarithmic power spectral density (LPSD) in the delta (1–4 Hz), theta (4–8 Hz), alpha (8–12 Hz), beta (12–30 Hz) and gamma (30–40 Hz) bands was computed for each subject, condition and channel using a fast Fourier transform with a Hanning-tapered window (EEGLAB).

We estimated broadband signal complexity using the Lempel-Ziv lossless compression algorithm applied to binary time series obtained from a median split of the instantaneous signal envelope (obtained via Hilbert transform) after Z-score conversion [43, 45, 47]. By definition, the median split resulted in the same proportion of 1’s and 0’s across all channels, thus avoiding biases due to unbalanced sequences.

### Local-global auditory stimulation paradigm

The Local-Global paradigm consists of auditory stimulation designed to evaluate responses to violations of local and global regularities, the latter being considered a signature of conscious information processing. Following Bekinschtein et al. [51], trials consisted of 5 brief sounds (50 ms each) separated by 100 ms. The first 4 sounds were identical, either with high (1600 Hz, “H”) or low (800 Hz, “L”) pitch. The final sound of the sequence could be identical to the others or different, leading to local standard (LLLLL, HHHHH) and local deviant (LLLLH, HHHHL) trials, respectively. Each block consisted of a global standard and a global deviant; importantly, the global deviant could be a local standard and vice versa. For each block, the global standard was repeated 4 times for habituation, then 4–7 global deviants were delivered interleaved with global standards, so that at least two global standards preceded each global deviant. Within each block, trials were randomly separated with silent intervals lasting 1350 to 1700 ms (in steps of 50 ms) and blocks were separated by 15 s. Finally, for each combination of global standard and deviant, blocks were repeated 5 times.

For the ERP analysis, EEG data was epoched and preprocessed following a similar procedure to that outlined in the previous section. Trials were segmented from −200 ms to +1300 ms relative to the first sound in the sequence. EEGLAB automated rejection (with the same criteria as used for the resting state data) was applied. The trials were then averaged in synchrony with stimulus onset, transformed to an average reference, band-pass filtered (0.5–20 Hz) and corrected for baseline over the 200 ms window before the onset. ERPs (P300) corresponding to local and global deviants were constructed for the drug and the placebo conditions, and compared against the baseline using Wilcoxon’s tests. An uncorrected threshold of 10 consecutive samples with *p* < 0.05 was implemented to determine which channels showed larger amplitudes of the global compared to the local deviants. For these channels, the same approach and threshold was used to assess differences between the active dose and the placebo condition.

### Physical activity

The Fitbit Charge 4 wristband was used to determine two measures of physical activity during each day of the experiment: the total displacement (in kilometers) and the number of steps taken during the day. Fitbit’s tracking of step counts was shown to be accurate by direct comparison with estimates based on video recordings [52]. A systematic review showed that these metrics are among the most accurate provided by the wristband [53].

### Statistical analyses

Results from both conditions (active dose and placebo) were compared using non-parametric paired Wilcoxon signed-rank tests or Whitney–Manney *U* tests in the case of unpaired data. Results without correction for multiple comparisons are reported when *p* < 0.05, and it is also indicated whether they remain statistically significant when adopting the Bonferroni correction for multiple comparisons, explicitly stating the number of comparisons. We did not estimate statistical power since we did not know a priori what effect size could be expected for the tasks and conditions of the experiment. Chi^2^ squared tests were applied to the contingency tables to determine whether participants were breaking the blind, both after the first and second measurement week. These tests were applied as implemented in Python’s *scipy* library (https://scipy.org).

Frequentist methods were complemented using Bayesian statistics to compare the evidence in favor of the null hypothesis with that in favor of the alternative hypothesis. We computed the Bayesian statistic BF10 (Bayes factor in favor of the alternative hypothesis over the null hypothesis) using the Jeffrey-Zellner-Siow (JZS) prior as implemented in Python’s *pingouin* library (https://pingouin-stats.org).

In the figures, all boxplots extend from the lower to upper quartile values with a line at the median; the whiskers extend from the upper/lower quartiles up to 1.5 times the interquartile range. Individual subjects are represented with single points scattered on top of the boxplots.

### Chemical characterization of samples

Each participant consumed a total of 1 g of dried *Psilocybe cubensis* for the active condition, separated in two doses of 0.5 g. Samples were dried at *≈*28° and ground into a fine powder, with different parts of the mushrooms homogeneously distributed. In total, there were three independent sources for the mushrooms that were consumed in the context of this experiment. Samples of 150 mg were collected from these sources and sent for chemical analysis to the Laboratory of Forensic Analysis of Biologically Active Substances, University of Chemistry and Technology Prague, Czech Republic. The quantification of alkaloids (psilocybin, psilocin, baeocystin and norbaeocystin) in the samples was performed by MK and KH using liquid chromatography–mass spectrometry (LC-MS), determining the following concentrations: psilocybin (640.2 μg/g), psilocin (950.7 μg/g), baeocystin (50.4 μg/g), norbaeocystin (12.5 μg/g).

## Results

### Unblinding of the experimental condition

Participants correctly unblinded the experimental condition in 49 of the 64 measurement weeks (75%). Of the 34 weeks corresponding to the active dose, 25 were correctly unblinded by the participants (73.5%), with a similar percentage for the placebo condition (70%). Chi^2^ tests showed that subjects did not break the blind during the first measurement week [Chi^2^(1) = 0.09, *p* = 0.76], but did break the blind for the second measurement week [Chi^2^(1) = 11.53, *p* = 0.0006], which could indicate that the information on the previous experimental condition facilitated the identification of the current condition.

### Acute effects

We first computed the sum of all items in the VAS to obtain an index of the overall intensity of the acute effects. VAS total scores were significantly higher for psilocybin vs. placebo for both dosing days (Fig. S1 of the supplementary material), prompting us to investigate whether this result was be driven by unblinding of the experimental condition. We divided these scores into two subsets, depending on whether the subjects correctly identified the active dose/placebo (“unblinded”) or failed to do so (“blinded”).

The VAS total scores are shown for Wednesday (first dosing day), Thursday, Friday (second dosing day) and Saturday in Fig. 1d, both for the “unblinded” (left) and “blinded” (right) subsets. Summary statistics and results of statistical tests are given in Tables S1 and S2 of the supplementary material. During both dosing days the participants reported VAS total scores significantly higher for the active dose vs. the placebo (corrected for multiple comparisons, *n* = 4); however, this difference was not present on days when a dose was not consumed, indicating the absence of carry-over effects. There were no significant differences in total VAS scores between the first and second dosing days. Increased VAS total scores were found in the “unblinded” subset (Fig. 1d, left) but not in the “blinded” one (Fig. 1d, right). These differences were supported by the Bayesian analysis, with BF10 values in the very strong (>30) range [54]. For the blinded group, BF10 values were between 3 and 1/3, which can be interpreted as insufficient evidence to settle between the alternative and null hypothesis, possibly due to the comparatively low sample size of the blinded group. For the blinded group, we observed a trend towards higher VAS total scores for psilocybin vs. placebo, but only for the first dosing day (Wednesday). It could be possible that after receiving the first dose and incorrectly identifying it as placebo, the participants would then receive the second dose already expecting a placebo, thus anticipating absent subjective effects.

Figure 1E presents the outcome of this analysis conducted separately for each VAS item. While differences were found for items related to imagination, dreamlike quality of the experience, spatial distortions and mind-wandering, these did not remain significant after applying the Bonferroni correction for multiple comparisons (*n* = 21). Except for four comparisons in the range of strong evidence (BF10 > 30, matching the significant differences indicated in Fig. 1E), the majority of the remaining BF10 values were inconclusive or close to inconclusive (between 1/3 and 3). Thus, we did not find robust evidence of consistent changes in the VAS sub-items across participants.

### Self-reported scales and questionnaires

**Fig. 2:**
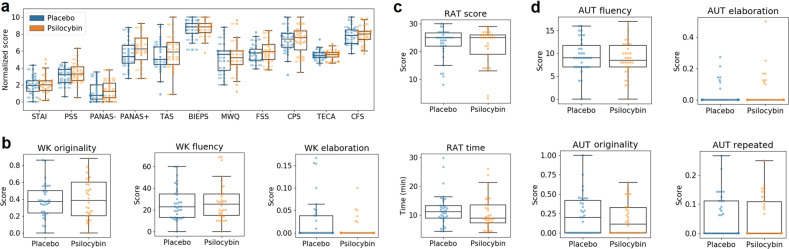
Results of the self-reported scales and questionnaires and creativity tests. **a** Boxplots showing the scores of all questionnaires for the active dose and placebo. **b** WK originality, fluency and elaboration. **c** RAT total score and time (in minutes). **d** AUT fluency, originality, elaboration and number of repeated answers. No significant differences were found at *p* < 0.05, uncorrected. STAI State Trait Anxiety Inventory, PSS Perceived Stress Scale, PANAS Positive and Negative Affect Scale, TAS Tellegen Absorption Scale, BIEPS Well-being Scale, MWQ Mind Wandering Questionnaire, FSS Flow State Scale, CPS Creative Personality Scale, TECA Cognitive-Affective Empathy Test, CFS Cognitive Flexibility Scale, RAT Remote Associates Test, AUT Alternative Uses Task, WK Wallach–Kogan Test.

We scored the scales and questionnaires included in Table 1 and summarized the results in Tables S1 and S2 of the supplementary methods. These tables include mean *±* SEM, *p*-values (Wilcoxon’s signed rank test or Whitney–Manney *U* tests) and BF10 values, both for all data considered together and for the “unblinded” and “blinded” data subsets). There were no significant differences between conditions at *p* < 0.05, uncorrected (except for the conscientiousness trait [*p* = 0.01], which did not remain significant after Bonferroni correction) and the majority of BF10 values were <1/3, indicating moderate evidence in support of the null hypothesis. Figure 2A presents boxplots of these scores for the complete dataset, with the exception of trait variables that were only assessed at baseline (i.e., BFI, STAI-T and SSS). Note that questionnaire scores were divided by their maximum possible value and multiplied by 10, yielding normalized scores to facilitate direct visual comparison. For the analysis restricted to the “blinded” subset, all BF10 values were between 3 and 1/3, indicative of insufficient evidence to settle between the alternative and null hypothesis.

### Creativity tests

We used the RAT to measure convergent creativity, scoring the total number of correct answers and the total elapsed time. To measure divergent creativity, we used the WK creativity test and the AUT. For the first, we scored the fluency (total number of words provided), the originality (responses that were given by only 5% of the group were considered unusual and scored only 1 point, responses that were given by only 1% of the group were considered unique and scored 2 points, other responses scored 0 points) and the elaboration, related to the amount of detail provided in each answer. The AUT was scored following these criteria, plus the number of repetitions in the answers. For the WK test and the AUT, higher scores are indicative of more creative performance (except for the repetition score). To avoid confounds due to different levels of fluency, the originality, repetitions and elaboration scores were normalized by the fluency score [55].

The results of this analysis are provided in Tables S3 and S4 of the supplementary methods and Fig. 2B–D. The tables include mean *±* SEM, *p*-values (Wilcoxon’s signed rank test or Whitney–Manney *U* tests) and BF10 values, both for all data considered together and for the “unblinded” and “blinded” data subsets). There were no significant differences between conditions at *p* < 0.05, uncorrected (except for the AUT fluency [*p* = 0.03] and the WK elaboration [*p* = 0.04] scores, which did not remain significant after Bonferroni correction) and all BF10 values were below 1/3 (except for the WK elaboration score, BF10 = 1.28), i.e., favoring the null hypothesis. For the analysis restricted to the “blinded” subset, all BF10 values were between 3 and 1/3, indicative of insufficient evidence to settle between the alternative and null hypothesis

### Perception and cognition

**Fig. 3:**
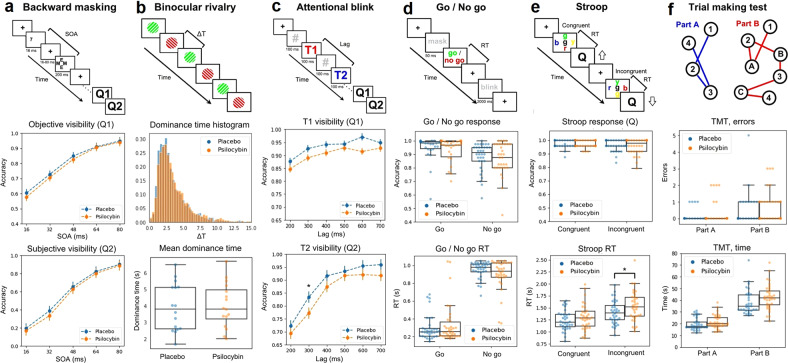
Results of tasks used to assess perception and cognition. The uppermost panel of each column contains a diagram of the task. **a** Backward masking. A target (number 7) is flashed and then masked; the time elapsed between target and mask is the stimulus onset asynchrony (SOA). Participants were queried for objective (Q1, comparison with number 5) and subjective (Q2) visibility. The panels below show the objective and subjective accuracy vs. SOA. **b** Binocular rivalry. Two different gratings were presented to each eye, resulting in alternation of the perceived grating, with the duration of the percept before switching being given by *∆*T (dominance time). The panels below show the histograms of *∆*T values and a comparison of <∆T> between conditions. **c** Attentional blink. A sequence of digits (separated by 100 ms) contains two target numbers (T1, T2), participants were required to identify both targets (Q1, Q2). Below, the rate of correct answers to Q1 and Q2. The visibility of the second target for lag 300 ms was reduced in psilocybin vs. placebo (*p* < 0.05, uncorrected). **d** Go / No Go. A mask is presented, followed by instructions to press a key (“Go”) or pass (“No Go”), registering accuracy and response times (RT). These two variables are shown in the panels below. Response times were slower for psilocybin vs. placebo (*p < 0.05, uncorrected). **e** Stroop. Participants were presented with the name of a color written in either the same (congruent) or different (incongruent) color, and asked to identify the color of the word (Q). The next two panels show the accuracy and the RT. **f** The participants were asked to join with lines dots with numbers (part A) or with alternating numbers and letters (part B), in both cases in increasing order. The remaining two panels show the rate of errors for each part of the task.

Next, we investigated whether microdosing with psilocybin mushrooms modulated perception and cognition across several tasks. The results are summarized in the different columns of Fig. 3. The uppermost panel of each column illustrates the contents and durations of the different screens shown to the participants, together with other relevant elements that are defined in the caption. We investigated the following metrics for each of the tasks: (a) objective and subjective accuracy vs. stimulus onset asynchrony (SOA) for backward masking, (b) average dominance durations (obtained by fitting a gamma function to the individual histograms) for binocular rivalry, (c) visibility rate of the first (T1) and second (T2) target as a function of the lag between them for the attentional blink, (d) response accuracy and response times (RT) for Go / No Go, (e) same variables for the Stroop test, (f) total errors and the time required to complete the task for the trial making test. Overall, we found decreased visibility of the second target with 300 ms lag in the attentional blink task, and increased RT in the Stroop task, both significant at *p* < 0.05 (BF10 > 1) but only without correction for multiple comparisons. We also found a significant increase in the time required for part A of the TMT test.

### Resting state EEG and Local-Global ERPs

**Fig. 4:**
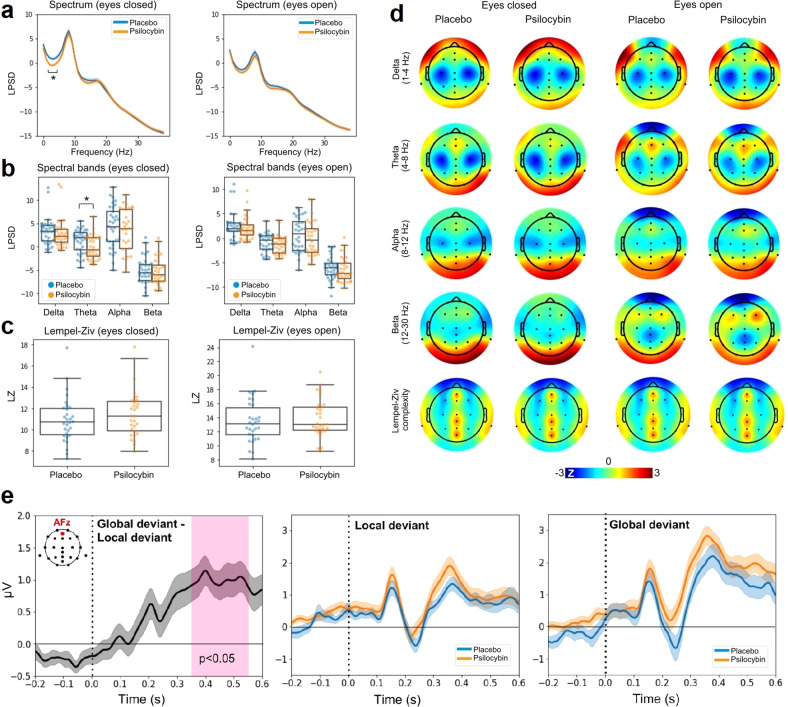
Results of the resting state EEG analysis. **a** LPSD vs. frequency (averaged across all channels) for eyes closed (left) and open (right), both for the placebo and the active dose (mean *±* SEM). **b** Same as in panel A, but binned using the following bands: delta (1–4 Hz), theta (4–8 Hz), alpha (8–12 Hz) and beta (12–20 Hz). **c** Global Lempel-Ziv complexity computed using the broadband EEG signal, compared between psilocybin and placebo conditions for eyes closed and open. **d** Topographic distribution of spectral power and Lempel-Ziv complexity for all combinations of active dose, placebo, eyes open and closed. **e** Results of the Local-Global ERP analysis. Left: global deviant minus local deviant ERPs located at AFz for the the placebo condition. Center: local deviant at AFz for placebo vs. psilocybin. Right: same as in panel C but for the global deviant. All ERP plots show mean *±* SEM. The vertical dashed lines coincide with the timing of the last sound in the trial. *p < 0.05 for the comparison between placebo and active dose (corrected for multiple comparisons).

Figure 4 presents the results of the resting state EEG analysis. Panel A displays the LPSD vs. frequency (averaged across all channels) for eyes closed (left) and open (right), both for the placebo and the active dose. As expected, the power spectra for eyes closed show a peak close to 10 Hz, which is attenuated in the eyes open condition. The psilocybin mushroom microdose resulted in decreased power in the theta range (4 to 8 Hz). Figure 4B presents the same results binned into four major frequency bands: delta (1–4 Hz), theta (4–8 Hz), alpha (8–12 Hz) and beta (12–20 Hz). Consistent with the spectra shown in Fig. 4A, only the eyes closed theta band power decreased under the active dose compared to the placebo (*p* < 0.05, both uncorrected and Bonferroni corrected with *n* = 4). Figure 4C compares the global Lempel-Ziv complexity computed using the broadband EEG signal between psilocybin and placebo conditions for eyes closed (left) and open (right). Figure 4D presents the topographic distribution of spectral power and Lempel-Ziv complexity for all combinations of active dose, placebo, eyes open and closed.

We computed the ERPs associated with local and global deviants from the Local-Global auditory stimulation. Across all channels, we investigated whether the global deviant resulted in larger late amplitude deflections compared to the local deviant, as expected from the previous literature [51]. Also consistent with previous work, the central-frontal channel AFz presented the largest effects. Figure 4E (left) shows the global deviant minus the local deviant ERPs located at AFz for the placebo condition; clearly, after 300 ms the global deviant presents a more sustained amplitude, which has been linked to conscious information processing. The center and right panels of Fig. 4E show the ERPs at AFz for local and global deviants, respectively, and for the active dose and placebo conditions. No significant differences (uncorrected) between conditions were found.

### Physical activity

**Fig. 5:**
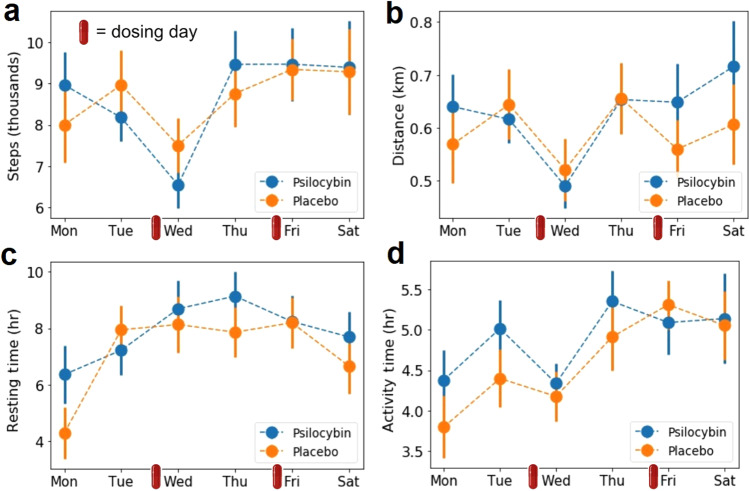
Results of the physical activity analysis. **a** Step count per week day. **b** Distance traveled per week day. **c** Resting time per week day. **d** Total activity time per week day. All plots show mean *±* SEM. The small red capsules indicate the dosing days.

Figure 5 summarizes the results of the physical activity analysis based on data provided by the Fitbit Charger 4 wristband. Figure 5A–D show the step count, distance traveled, resting and activity time, respectively, per each day of the week during the experiment. We did not find differences (uncorrected) between the active dose and placebo conditions. The decrease in activity on Wednesday that is seen for step count, distance traveled and activity time may occur because several measurements requiring to be seated were conducted on that day.

## Discussion

According to our results, 0.5 g of dried mushroom material did not present significantly positive impact on creativity (divergent and convergent thinking), cognition, physical activity levels, and self-reported measures of mental health and well-being. However, we observed a trend towards impaired performance in some cognitive tasks (i.e., attentional blink and Stroop). In contrast, the overall acute effects induced by the microdose (VAS total score) were significant, although they lacked consistency across participants. We also found decreased EEG power in the theta band under psilocybin, which is consistent with the broadband spectral power reductions reported for higher doses.

Ample anecdotal evidence suggests that microdosing can improve mood, well-being, creativity, and cognition [17, 28], and recent uncontrolled, open-label observational studies have provided some empirical support for these claims [7–9, 18–20, 27–29]. While encouraging, these studies are vulnerable to experimental biases, including confirmation bias and placebo effects [56]. This is especially problematic in the case of microdosing, since users make up a self-selected sample with optimistic expectations about the outcome of the practice [4, 57]. This positivity bias, combined with the low doses and self-assessment of the drug effects via scales and questionnaires, paves the way for a strong placebo response.

To date, we could identify relatively few human studies of microdosing with psychedelics following a rigorous experimental design. The first was conducted by Yanakieva and colleagues, who investigated three comparatively low doses of LSD (5, 10, and 20 µg) [34], concluding that LSD affected the estimation of time intervals, without other significant changes in perception, mental processes and concentration. However, the researchers did not assess the preexisting motivations and expectations of the participants, and the laboratory setting of the experiment might have contributed to their suboptimal performance. Bershad and colleagues investigated an inactive placebo and three different doses of LSD (6.3, 13, and 26 µg) separated by 1-week intervals [33]. At the highest dose, the drug increased ratings of vigor and slightly decreased positivity ratings of images with positive emotional content. Measurements of mood, cognition, and physiological responses did not show differences between conditions. Another study by the same group [37] showed that a low dose of LSD (13 µg) increased amygdala seed-based connectivity with the right angular gyrus, right middle frontal gyrus, and the cerebellum, and decreased amygdala connectivity with the left and right postcentral gyrus and the superior temporal gyrus. Although this dose of LSD had weak effects on mood, they were positively correlated with the increase in amygdala–middle frontal gyrus connectivity strength. Family et al. established the safety of LSD microdosing in older volunteers, but did not report substantial positive effects [36]. Hutten and colleagues reported dose-dependent positive effects on mood, but also anxiety and cognitive impairment [35]; also, the same group showed that low doses of LSD can increase brain-derived neurotrophic factor blood plasma levels in healthy volunteers [58]. Finally, both Szigeti et al. [26]. and Van Elk et al. [38]. combined double-blind placebo-controlled design with field measurements under natural conditions. Both found positive effects of microdosing on the primary outcome of their respective studies; however, these results could be explained by breaking of the placebo condition. In particular, Van Elk et al. found that more than 60% of the participants were breaking blind to the experimental condition [38], consistent with the unblinding rate found in our study (75%).

Our results add to this series of double-blind placebo-controlled studies questioning the validity of anecdotal evidence for microdosing [4]. In comparison to previous studies [37, 38], most results remained negative even when the statistical analyses were restricted to measurements obtained from unblinded subjects (with the exception of the VAS total scores of acute effects, see Fig. 2A). We note, however, that Bayesian statistics suggested insufficient sample size for the blinded group, a limitation to be overcome by future studies. Overall, few uncorrected differences were found. In the case of tasks to assess cognitive function, these differences were indicative of impaired performance, which is consistent with previous experiments [35] and with the observation that higher doses of serotonergic psychedelics negatively affect cognitive functions such as attention and decision making [59]. It has also been suggested that psychedelics might facilitate visual perception by increasing the broadband of consciously perceived information [59]. This was supported by studies of binocular rivalry, showing that two doses of psilocybin (115 μg/kg and 250 μg/kg) slowed down the rate of binocular rivalry switching and increased the proportion of reports of mixed percepts [60, 61]. Our study failed to replicate these findings, possibly due to the lower effective dose of psilocybin contained in the mushroom preparations. Also, we directly investigated the potential influence of microdosing on conscious perception using a backward masking paradigm (for visual perception) [62] and the Global-Local paradigm combined with EEG for ERP analysis of global and local deviants (for auditory perception) [51]. Neither of these tasks revealed a significant effect of *Psilocybe cubensis* microdosing on conscious information processing.

We found reduced power of EEG theta oscillations during the effects of the psilocybin microdose, heralding the larger broadband reductions observed for higher doses [49]. However, the analysis of Lempel-Ziv complexity failed to reveal differences between conditions, suggesting that increased signal entropy could constitute a specific signature of the altered consciousness elicited by psychedelics or other non-pharmacological mechanisms [63–65]. Reduced vigilance is a potential non-pharmacological mechanism underlying the observed changes in theta power. Given that theta power is increased as vigilance is reduced, the result would be consistent with participants becoming drowsy under the placebo, while maintaining alertness under the active dose [66]. This explanation is consistent with a slightly stimulant effect of the psilocybin microdose; however, changes in vigilance would be expected to affect other frequency bands as well, and this was not observed in the data.

Daily levels of physical activity constitute a proxy of the potential effects of microdosing on mood and well-being. The relationship between physical activity and mental health is well-established [67] and has been adopted as a marker of treatment efficacy for depression [68]. Currently, the potential association between changes in physical activity levels and psychedelic use remains unexplored. While our results did not reveal an effect of microdosing on this domain, future studies could further this investigation using higher doses of serotonergic psychedelics, both in healthy and clinical populations [25], and conducting measurements over longer time periods.

While the study of microdosing with *Psilocybe cubensis* mushrooms presents advantages in terms of ecological validity, it also raises problems associated with unknown or inconsistent chemical composition. We analyzed the contents of three samples pooled together, estimating an effective dose of *≈*0.9 mg of psilocybin; however, this dose could have been higher or lower depending on the source of the mushrooms consumed by each participant. Also, we did not correct the effective psilocybin dose using the weight of the participants. While this adjustment might not be necessary for larger doses, its importance for microdosing remains unexplored [69]. The amount of psilocybin/psilocin found in our samples is within the expected values for the mushrooms or truffles that are consumed in the context of microdosing [8, 26, 38]; in particular, it is almost identical to the values reported by Prochazkova et al. [18]. Nevertheless, other recent studies used truffles with higher concentrations of psilocin and psilocybin; for instance, Van Elk and colleagues investigated the effects of 0.7 g of psilocybin-containing truffles, with an estimated amount of 1.5 mg of psilocybin per dose. As acknowledged by the authors of this study, 0.7 g exceeds what is frequently considered the upper limit when microdosing with psilocybin mushrooms (note, however, that what constitutes “microdosing” is not precisely defined) [25]. It is also important to consider the possibility that our samples lost potency between the experiment and their chemical analysis. As shown by Gotvaldová and colleagues, the concentration of psilocybin can drop up to 50% during the first months of storage [70], which in our case would imply original concentrations similar to those reported by Van Elk and colleagues. Finally, our samples contained small amounts baeocystin and norbaeocystin; whether these compounds are psychoactive in humans is still under discussion [71].

It is possible that the experimental design of this study was not optimal to detect some of the claimed positive effects of microdosing. We investigated the effects of two doses per week, yet microdosing is generally conducted over extended periods of time according to an ample variety of dosing schedules [17]. By design, our study could not assess the cumulative effects of microdoses consumed over periods of several days. Instead, we decided to include time-consuming assessments which required active participation of the research team (e.g., computer-based cognitive tasks, EEG), which were too disruptive to be repeated routinely for an extended period of time, but at the same time addressed comparatively understudied potential effects of microdosing. Due to the build-up of tolerance after repeated administration of serotonergic psychedelics [11], we speculated that the intensity of these effects could only decrease in time; further motivating our focus on the acute effects of microdosing instead of its potential cumulative effects. This decision was also based on the variability of microdosing schedules adopted by users and represented in the current literature [25], which raises the concern of schedule-specific results as an obstacle to address the consistency of findings between studies. Future research should explore whether the positive effects of microdosing can be selectively enabled or facilitated by certain long-term dosing schedules. Also, this study was conducted in healthy participants, and thus the lack of significant findings could stem from ceiling effects. It remains possible that microdoses of psilocybin mushrooms exert positive effects on cognition and mental health, but only in populations of patients already suffering from impairments in these domains.

In conclusion, we conducted a controlled study of microdosing in individuals who were already planning to start their own microdosing protocol. While small amounts of dried *Psilocybe cubensis* mushrooms reliably induced significant subjective effects, their impact in other domains was negligible or even indicative of impaired performance. Clearly, more research is needed to decide whether microdosing with psychedelics can deliver at least some of its promised positive effects. This future research should also explore the potential impact of microdosing on aspects of human physiology that could compromise its long-term safety; for instance, by addressing the potential consequences of chronic 5-HT_2B_ receptor stimulation on the health of the circulatory system, among other important points [25, 72]. Until this research is conducted, it remains impossible to ascertain that long-term microdosing is a safe practice with desirable effects, and to rule out that these effects arise as a consequence of expectation or confirmation biases.

## Supplementary information

Supplementary material

## Data Availability

Documented data files used in this manuscript are available at the Open Science Framework (OSF) project page https://osf.io/hnxq6/.
