# Supplementary material for: Microdosing with psilocybin mushrooms: a double-blind placebo-controlled study
Source: Transl Psychiatry. 2022 Aug 2;12:307. doi: 10.1038/s41398-022-02039-0 (PMC9346139; doi:10.1038/s41398-022-02039-0)
Supplement: Supplementary file 1 — Supplementary material [file 41398_2022_2039_MOESM1_ESM.pdf]

Supplementary materials

Recruitment process

Interested individuals got in contact with the researchers by email. This first contact was followed by a phone conversation with the candidate participants, who were briefed on the details and objectives of the experiment, as well as on the inclusion and exclusion criteria. They received a copy of the informed consent form together with a detailed written explanation of the experiment and its objectives, with a detailed explanation of the need for a blinded and randomized control condition with an inactive placebo. After signing the form, all subjects underwent a psychological interview to screen for the exclusion criteria that are detailed below. Afterwards, participants and researchers agreed on the start of the microdosing procedure according to the schedule presented in the next session.

Selection criteria

Participants were planning to start a microdosing protocol with their own *Psilocybe cubensis* material at the time they enrolled in this study. As a condition to be included, they were asked to follow a pre-arranged dosing schedule, to abstain from consuming psychoactive drugs (including alcohol and caffeine) during the study weeks, and to avoid eating three hours before consuming each microdose. A non-diagnostic psychiatric interview was conducted according to the guidelines by Johnson et al. (2008). Subjects who fulfilled DSM-5 criteria for the following disorders were excluded from the experiment: schizophrenia or other psychotic disorders, and type 1 or 2 bipolar disorder (both also in first and second degree relatives), substance abuse or dependence over the last 5 years (excluding nicotine), depressive disorders, recurrent depressive episodes, obsessive-compulsive disorder, generalized anxiety disorder, dysthymia, panic disorder, bulimia or anorexia, as well as subjects with history of neurological disorders. Subjects under psychiatric medication of any kind were also excluded.

Experimental setting

All experiments were conducted in a comfortable house which hosted only the researchers and the participant, with separate rooms fitted for the needs of each task. Environmental sounds were kept to a minimum and participants were provided with noise-canceling headphones in case they were

considered necessary. During EEG recordings, the main power line was interrupted in order to avoid artifacts due electrical currents in the proximity of the electrodes. To avoid the possibility of subjects driving under the effects of psilocybin, all subjects were taken by car to the experimental premises and then back to their points of departure

VAS items

During the acute effects, subjects used a VAS to provide their score for the following items: “My imagination was extremely vivid”, “The experience had a dreamlike quality”, “Sounds influenced things I saw”, “My sense of space and size was distorted”, “I felt unusual bodily sensations”, “My thoughts wandered freely”, “My perception of time was distorted”, “I saw geometric patterns”, “Edges appeared warped”, “My thinking was muddled”, “I saw movement in things that weren’t really moving”, “I experienced a sense of merging with my surroundings”, “Things looked strange”, “I felt like I was floating”, “The experience had a supernatural quality”, “I experienced a disintegration of myself or ego”, “I felt a profound inner peace”, “The experience had a spiritual or mystical quality”, “I felt afraid”, “I feared losing control of my mind”, “I felt suspicious and paranoid”.

The choice of VAS to measure the subjective acute effects (as well as the VAS items included for this purpose) was based on several previous studies of LSD, psilocybin, and other serotonergic psychedelics, where significant correlations with neuroimaging data was reported. We considered it important to include a measure of the subjective acute effects given that some of these effects (e.g. visual distortions, changes in the perception of bodily boundaries, alterations in mood, etc.) are characteristic of larger doses of psychedelics, and thus might contribute to breaking the blinding of the experimental conditions.

Blinding procedure

The complete blinding procedure consisted of the following steps:

1. Two gel capsules were filled with 0.5 g of dried and finely ground and homogenized active material, yielding two doses of 0.5 g each. These capsules were stored in an airtight plastic bag

within a paper envelope. For each independent source of mushrooms, samples of 150 mg were taken and preserved for chemical analysis.

2. This process was repeated using the same number of capsules, but each filled with 0.5 g of dried and ground edible mushrooms (e.g. *Suillus granulatus*). These capsules were stored in an airtight plastic bag within paper envelopes identical to those used in the previous step.

3. The blinded conditions could be identified by a folded paper with a code that was introduced to both envelopes.

4. Subjects took the envelopes and randomly selected one of them at the beginning of the experiment, leaving the other for the second week.

5. After the data was collected, scored and analyzed, subjects reported to the experimenters the code corresponding to each of their envelopes. Next, the third party in charge of the blinding used these codes to determine which envelope corresponded to each condition, sharing this information with the researchers to perform the final statistical analyses. In turn, the order of the conditions was also revealed to the participants by the researchers.

Measurements per day of the week

Baseline measurements of psychological traits were conducted on the first day of the week for each condition. Also, participants received and started wearing the Fitbit Charge 4 wristband to track their daily levels of physical activity. On all the days of the protocol, subjects also completed a self-reported scale to assess the subjective effects perceived during the day, accessed through a link that they received via the mobile messaging app Telegram.

Capsules (active or placebo, depending on the week) were consumed on Wednesday (first dosing day) and Friday (second dosing day). On the same days, the participants visited the experimental premises and performed a series of measurements that are detailed in one of the following sections. Measurements started 1.5 h after consuming the capsule (Passie et al., 2002).

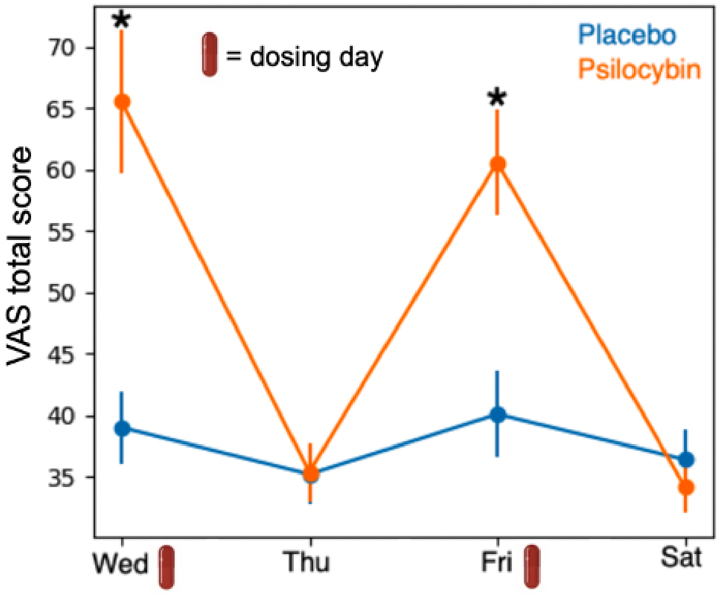

Figure S1. VAS total score (meanSEM) per condition, from Wednesday (first dosing day of the week) to Saturday (last day of the experiment), for all participants.
*p<0.05, Bonferroni corrected (n=4).

Self-reported scales and questionnaires

Big Five Inventory (BFI). A validated Spanish version of the inventory assessing five dimensions of personality: neuroticism, extraversion, openness to experience, agreeableness, and conscientiousness (Benet-Martinez and John, 1998). The BFI questionnaire consists of 44 items based on a 5-point Likert scale. Multiple studies suggest that psychedelics are capable of inducing short and long-term changes in personality (Bouso et al., 2018). Moreover, some of these changes (e.g. increased openness) might be contributing factors to the therapeutic effects of psychedelics, as well as to the long-term positive changes in subjective well-being reported by healthy individuals.

Short Suggestibility Scale (SSS). An inventory that assesses suggestibility, created by Kotov et al. (2004), and translated to Spanish by the authors. The questionnaire consists of 21 items based on a 5-point Likert scale. It has been shown that psychedelics can enhance suggestibility in healthy volunteers, which points towards the relevance of investigating whether microdosing can induce a similar effect (Carhart-Harris et al., 2015). This is also important in the context of

potential use of psychedelic microdoses as adjuncts to psychotherapy, where suggestibility plays a relevant role.

Tellegen Absorption Scale (TAS). A 34-item scale developed to measure the capacity of an individual to become absorbed in the performance of a task (Tellegen and Atkinson, 1974), translated to Spanish by the authors. This psychological construct is positively correlated with the overall intensity of the effects elicited by psychedelic drugs, and also implicated in some of its most intriguing effects, such as the induction of spiritual or mystical experiences (Haijen et al., 2018).

Multiple mental health indicators were chosen to assess some of the benefits attributed to microdosing in anecdotal reports, and have been included in previous studies of microdosing and its effects in healthy individuals. These include the State-Trait Anxiety Inventory, the Positive and Negative Affect Schedule (PANAS), Perceived Stress Scale (PSS), and the Psychological Well-being Scale (BIEPS). We also included the Mind Wandering Scale (MWQ), given the suggestion that mind wandering negatively contributes to psychological well-being, and considering the hypothesis that serotonergic psychedelics might act by reducing activity levels in areas recruited during mind wandering and self-referential thought, resulting in a state which shares similarities with those achieved by means of mindfulness or other forms of meditation (Carhart-Harris, 2018).

State-Trait Anxiety Inventory (STAI-T / STAI-S). Validated Spanish editions of commonly used scales which measure state anxiety (situational anxiety of a temporary nature) and trait anxiety (stable trait linked to individual characteristics) (Spielberger et al., 1983). The instrument comprises 40 items and is based on a 4-point Likert scale.

Positive and Negative Affect Schedule (PANAS). A validated Spanish version of a psychometric scale that has been widely used to measure dimensions of affect, both positive and negative (Watson et al., 1988). The instrument consists of 20 affirmations based on a 5-point Likert scale.

Perceived Stress Scale (PSS). Validated Spanish version of an instrument to assess how different situations affect the feelings and perceived stress of the respondents, consisting of 10 affirmations based on a 5-point Likert scale (Cohen et al., 1994).

Psychological Well-being Scale (BIEPS). A scale used to measure eudemonic well-being in adults (including dimensions of acceptance, perception of control, social ties, and autonomy and projects) (Castro et al., 2002). It consists of 13 questions based on a 3-point Likert scale. Originally developed in Spanish.

Mind Wandering Scale (MWQ*)*. A 5-question instrument developed by Mrazek et al. (2013) to measure mind-wandering trait levels. It is a 6-point Likert-type scale, translated to Spanish by the authors.

Cognitive-Affective Empathy Test (TECA). Developed in Spanish by López-Pérez et al. (2008), TECA is a test that aims to measure both components that empathy is comprised of. The test has 33 items in a 5-point Likert scale. A positive relationship between the effects of psychedelics and empathy (including in low or sub-acute doses) has been proposed.

Cognitive Flexibility Scale (CFS). A scale used to measure three factors of cognitive flexibility (Martin & Rubin, 1995), translated to Spanish by the authors. It has 12 items in a 6-point Likert-type scale.

Creativity tests

We assessed the Cognitive Flexibility Scale (CFS), Creative Personality Scale (CPS), Flow State Scale (FSS), and creativity tests to measure convergent/divergent thinking: Remotes Associates Test (RAT; convergent thinking), the Alternative Uses Task (AUT; divergent thinking), and the Wallach-Kogan Test (WK; divergent thinking). The inclusion of these measures responds to several anecdotal reports of enhanced creativity due to microdosing, as well as previous studies reporting mixed evidence in favor of this enhancement.

Remotes Associates Test (RAT). Spanish version of the test developed by Mednick & Mednick (1959), widely used to measure creative convergent thinking. The subject is given three words that appear to be unrelated and must think of a fourth word that is related to the previous ones.

Alternative Uses Task (AUT). Spanish version of the test to assess divergent thinking and creativity. Subjects were asked to think of and write of as many uses as possible for a simple item during two minutes (Guilford, 1967).

Wallach-Kogan Test (WK). Spanish version of the test to assess divergent thinking and creativity. Subjects were asked to come up with as many items as possible within a certain general group, without time constrains (Wallach and Kogan, 1965).

Creative Personality Scale (CPS). The scale is aimed to assess creative behavior and creative personality traits (Gough et al., 1979). The instrument consists of 21 affirmations based on a 4-point scale, translated to Spanish by the authors.

Flow State Scale (FSS). Developed by Jackson & Marsh (1996), the FSS is a 36- item scale, rated on a 5-point Likert-type scale, that measures nine different dimensions of the flow state. A validated Spanish version was administered to the participants.

Tasks to measure perception and cognition

We included computer-based tasks to investigate the effects of microdosing in the following domains: binocular rivalry (visual perception), backward masking (conscious visual perception), trail making test (attention and coordination), Go / No Go (inhibitory control), attentional blink (attention), and the Stroop test (inhibition). We included these measures as a basic characterization of the effects of psilocybin microdosing on cognition, given the conflicting claims of cognitive function enhancement caused by psychedelic microdoses vs. the observation that "standard" doses can impair at least some cognitive functions. We also included the Local-Global paradigm to evaluate EEG responses to violations of local and global regularities, the latter considered a signature of conscious information processing (Bekinschtein et al., 2009).

Binocular rivalry (BR). A pair of superimposed circles with opposite 45° gratings were presented within 5.4° of the fixation cross. When viewed through red and green filter glasses, each of the gratings was presented to one of the participant eyes. Subjects were instructed to maintain fixation and to report changes of the dominant stimulus by pressing a key, lasting for a total of 10 minutes. Previous reports have shown that the alternation rate between dominant stimuli is modulated by psilocybin (Carter et al., 2007; Carter et al., 2010). This task is outlined in the first row of Figure 3.

Backward masking (BM). Task used to study conscious visual perception, adapted from Del Cul et al. (2007). A digit between 0 and 9 (known as “target”) was briefly flashed (17 ms) at the left/right of the former location of the fixation cross (the location is randomly chosen) using different levels of contrast with the background. After a variable delay, known as stimulus onset asynchrony (SOA), a mask consisting of four letters appeared surrounding the former location of the target. Subjects were asked to indicate whether they perceived the target (subjective visibility report) and then to compare its magnitude relative to number 5 (objective visibility report). First, subject-specific initial contrast values were determined using a staircase procedure, in which the contrast was decreased (i.e. the task was made harder) every time the subjective visibility report was correct, and vice versa. The final contrast was computed as the mean over the 18 last reversals. Afterwards, the subject performs the task with the contrast value found in the staircase procedure with SOA, randomly using the values 16, 32, 478, 64 and 80 ms for the SOA. The performance is given by the objective and subjective visibility accuracy vs. SOA. This task is outlined in the first row of Figure 3.

Trail Making Test (TMT). A widely used test to assess functions such as attention, flexibility, speed and visuomotor integration. Performance is evaluated using two different tracking conditions: Part A involves connecting numbers from 1 to 25 in ascending sequence, while Part B involves connecting numbers and letters in alternating and ascending order (Reitan & Wolfson, 1993). This task is outlined in the first row of Figure 3.

Go / No Go (GNG). This task is designed to measure response inhibition. Subjects were asked to respond as quickly as possible to a 'go' stimulus (string "SSSTSSS") while avoiding to respond to a 'no go' stimulus (string "SSSHSSS"). Before presentation, both strings were briefly masked with numerals (i.e. '#######') during 50 ms. The performance was assessed in terms of the response time (RT) and the normalized accuracy (number of trials with correct responses divided by 200). This task is outlined in the first row of Figure 3.

Attentional Blink (AB). Used to measure the refractory period that follows the successful detection of a target in an attention demanding task (Shapiro et al., 1997). On each trial, a fixation cross was presented in the center of a laptop screen for a duration randomly selected between 1000 and 1500 ms. Each fixation cross was followed by a stream of serial visual stimuli consisting of 10–21 black letters, each presented for 100 ms and randomly selected from the alphabet with the following two restrictions: 1) pairs of successive letters could not be the same and 2) letters I, O, Q, S, X and Z were excluded. Two of the letters of the sequence were replaced by red digits between 2 and 9, which corresponded to targets T1 and T2. Targets were separated by lags ranging for 1 position in the sequence (100 ms) to 7 positions (700 ms), with T2 being presented 2 to 4 positions before the end of the stream. At the end of each trial, participants were instructed to detect digits T1 and T2, and their performance was determined by the rate of correct detections of each target. The task consisted of 140 trials, with the relevant parameters randomized across trials. This task is outlined in the first row of Figure 3.

Stroop Test (ST). A neuropsychological test used to assess the ability to inhibit cognitive interference (Stroop, 1935). At each trial, participants were shown a word corresponding to a color ("blue", "yellow", "red", "green"), with a color congruent (e.g. "yellow" shown in yellow) or incongruent (e.g. "yellow" shown in blue) with the word. At the center of the screen, the fixation point was surrounded by one word for each possible color (in randomized positions) and participants were prompted to select the stimulus color using the keyboard arrow keys. The experiment consisted of 50 trials, with the same proportion of congruent and incongruent word-color pairs. Performance in this task was assessed by the normalized accuracy and the RT. This task is outlined in the first row of Figure 3.

Summary of statistical analyses

See the Open Science Framework (OSF) project page (https://osf.io/hnxq6/) for additional files including p-values and BF10 values.

	**All (blinded+unblinded)**			
**Scale - Factor**	**Mean ± SEM**	**Mean ± SEM**	**p-value**	**BF10**
	**Psilocybin**	**Placebo**		
VAS (Wed)	65.52±5.84	38.97±2.95	7e-5	143.5
VAS (Thu)	35.32±2.41	35.14±2.42	0.83	0.25
VAS (Fri)	60.55±4.30	40.05±3.48	9e-5	52.86
VAS (Sat)	34.13±2.12	36.35±2.48	0.58	0.30
BFI - Extraversion	29.41±0.76	29.38±0.82	0.70	0.25
BFI - Agreeableness	35.02±0.68	35.02±0.82	0.93	0.25
BFI - Conscientiousness	29.76±0.78	28.14±0.89	0.01	0.36
BFI - Neuroticism	19.61±0.96	19.61±0.97	0.82	0.25
BFI - Openness	42.17±0.93	42.17±0.88	0.93	0.25
STAI-T	3.35±0.15	3.47±0.17	0.68	0.27
STAI-S	2.12±0.19	1.91±0.18	0.35	0.32
SSS	3.34±0.19	3.02±0.15	0.03	0.51
PANAS-	1.30±0.18	1.22±0.18	0.66	0.26
PANAS+	6.08±0.30	5.61±0.26	0.10	0.43
PSS	3.59±0.21	3.49±0.23	0.78	0.26
TAS	5.75±0.34	5.45±0.28	0.15	0.30
BIEPS	8.63±0.14	8.53±0.18	0.86	0.27
MWQ	5.27±0.33	5.20±0.30	0.62	0.25
FSS	5.93±0.22	5.69±0.17	0.30	0.33
CPS	7.20±0.26	7.24±0.23	0.84	0.25
TECA	5.54±0.09	5.50±0.09	0.30	0.26
CFS	7.87±0.17	7.80±0.17	0.92	0.25

Table S1. Results of the statistical analyses for self-reported scales and questionnaires (p-values obtained using paired Wilcoxon signed-rank tests, BF10 indicate the Bayes factor for the alternative hypothesis over the null hypothesis).

	Unblinded				Blinded			
**Scale - Factor**	**Mean ± SEM Psilocybin**	**Mean ± SEM Placebo**	**p-value**	**BF10**	**Mean ± SEM Psilocybin**	**Mean ± SEM Placebo**	**p-value**	**BF10**
VAS (Wed)	67.88±6.09	37.29±2.48	0.0002	83.36	59.00±4.93	43.00±3.75	0.16	0.70
VAS (Thu)	34.96±2.23	32.25±1.55	0.94	0.37	36.33±2.84	42.10±3.49	0.59	0.46
VAS (Fri)	63.72±3.94	34.75±1.83	2e-6	1e4	51.77±4.87	52.80±5.14	0.83	0.40
VAS (Sat)	32.96±1.73	35.66±2.42	0.54	0.36	37.44±2.87	38.00±2.60	0.90	0.40
BFI - Extraversion	29± 0.93	29.04 ± 0.99	0.98	0.28	30.55±1.35	30.20±1.57	0.86	0.40
BFI - Agreeableness	34.64± 0.81	35.04± 1.03	0.73	0.29	36.11±1.33	35± 1.42	0.56	0.45
BFI - Conscientiousness	29.04±1.01	28.62±0.94	0.54	0.29	29.88±1.11	27±2.12	0.34	0.66
BFI - Neuroticism	20.37±1.17	19.92±1.25	0.77	0.29	18.77±1.27	17.80±1.78	0.83	0.43
BFI - Openness	42.16±0.91	42.92±1.06	0.52	0.32	40.11±1.95	42.20±2.20	0.53	0.48
STAI-T	3.33±0.19	3.52±0.19	0.53	0.34	3.40±0.25	3.35±0.42	0.59	0.40
STAI-S	2.09±0.24	1.92±0.23	0.56	0.31	2.22±0.35	1.88±0.32	0.83	0.48
SSS	3.34±0.23	3.10±0.17	0.43	0.37	3.34±0.34	2.80±0.36	0.41	0.59
PANAS-	1.36±0.24	1.14±0.22	0.65	0.33	1.13±0.27	1.40±0.38	0.77	0.45
PANAS+	6.03±0.37	5.63±0.31	0.32	0.37	6.22±0.55	5.55±0.56	0.36	0.52
PSS	3.60±0.24	3.34±0.26	0.61	0.35	3.55±0.44	3.85±0.47	0.59	0.43
TAS	5.78±0.40	5.42±0.26	0.36	0.38	5.65±0.78	5.52±0.79	0.90	0.40

BIEPS	8.72±0.13	8.62±0.23	0.73	0.30	8.37±0.45	8.30±0.34	0.80	0.40
MWQ	5.36±0.38	4.95±0.32	0.58	0.37	5.02±0.72	5.80±0.72	0.51	0.49
FSS	5.97±0.29	5.73±0.20	0.52	0.34	5.81±0.28	5.61±0.37	0.51	0.43
CPS	7.16±0.29	7.22±0.24	0.99	0.28	7.32±0.65	7.30±0.60	0.77	0.40
TECA	5.54±0.11	5.36±0.09	0.24	0.50	5.53±0.19	5.82±0.21	0.56	0.58
CFS	7.96±0.18	7.83±0.21	0.73	0.31	7.61±0.46	7.73±0.29	0.99	0.41

Table S2. Results of the statistical analyses for VAS, self-reported scales and questionnaires, for participants who incorrectly/correctly unblinded the experimental condition (p-values obtained using Whitney-Manney U tests, BF10 indicate the Bayes factor for the alternative hypothesis over the null hypothesis).

	All (blinded+unblinded)			
**Test - Score**	**Mean ± SEM**	**Mean ± SEM**	**p-value**	**BF10**
	**Psilocybin**	**Placebo**		
RAT - Correct	21.88±1.12	22.94±0.93	0.22	0.31
RAT- Time (s)	651.88±57.07	715.73±56.78	0.25	0.32
AUT - Fluency	8.76±0.62	9.52±0.61	0.03	0.34
AUT - Originality	0.18±0.03	0.22±0.04	0.29	0.33
AUT - Repetitions	0.05±0.01	0.05±0.01	0.59	0.26
AUT - Elaboration	0.04±0.01	0.03±0.01	0.54	0.29
WK - Fluency	27.50±2.93	25.94±2.51	0.60	0.26
WK - Originality	0.39±0.04	0.37±0.03	0.83	0.26
WK - Elaboration	0.02±0.01	0.01±0.01	0.04	1.28

Table S3. Results of the statistical analyses for creativity tests: RAT, AUY, and WK (p-values obtained using paired Wilcoxon signed-rank tests, BF10 indicate the Bayes factor for the alternative hypothesis over the null hypothesis).

Test - Score	Unblinded<br>Mean ± SEM Psilocybin	Mean ± SEM Placebo	p-value	BF10	Blinded<br>Mean ± SEM Psilocybin	Mean ± SEM Placebo	p-value	BF10
RAT - Correct	22.4±1.38	23.20±1.09	0.81	0.31	20.44±1.97	22.30±1.95	0.68	0.47
RAT- Time (s)	679.52±74.03	741.62±74.82	0.41	0.32	575.11±75.04	653.60±80.39	0.34	0.48
AUT - Fluency	8.64±0.74	8.79±0.70	0.96	0.28	9.11±1.27	11.30±1.17	0.34	0.69
AUT - Originality	0.16±0.03	0.17±0.04	0.95	0.29	0.23±0.08	0.35±0.09	0.38	0.53
AUT - Repetitions	0.04±0.01	0.05±0.01	0.90	0.30	0.06±0.02	0.06±0.01	0.73	0.40
AUT - Elaboration	0.05±0.02	0.03±0.01	0.49	0.42	0.01±0.01	0.03±0.02	0.56	0.50
WK - Fluency	26.64±3.70	26.79±3.32	0.81	0.28	29.88±4.77	23.90±3.59	0.30	0.57
WK - Originality	0.39±0.05	0.36±0.02	0.81	0.32	0.39±0.03	0.07±0.02	0.96|	0.40
WK - Elaboration	0.03±0.01	0.01±0.01	0.19	0.81	0±0	0.02±0.01	0.09	0.58

Table S4. Results of the statistical analyses for creativity tests, for participants who incorrectly/correctly unblinded the experimental condition (p-values obtained using Whitney-Manney U tests, BF10 indicate the Bayes factor for the alternative hypothesis over the null hypothesis).

References

Bekinschtein, T. A., Dehaene, S., Rohaut, B., Tadel, F., Cohen, L., & Naccache, L. (2009). Neural signature of the conscious processing of auditory regularities. *Proceedings of the National Academy of Sciences*, *106*(5), 1672-1677.

Benet-Martínez, V., & John, O. P. (1998). Los Cinco Grandes across cultures and ethnic groups: Multitrait-multimethod analyses of the Big Five in Spanish and English. Journal of personality and social psychology, 75(3), 729.

Bouso, J. C., Dos Santos, R. G., Alcázar-Córcoles, M. Á., & Hallak, J. E. (2018). Serotonergic psychedelics and personality: A systematic review of contemporary research. *Neuroscience & Biobehavioral Reviews*, *87*, 118-132.

Carter, O. L., Hasler, F., Pettigrew, J. D., Wallis, G. M., Liu, G. B., & Vollenweider, F. X. (2007). Psilocybin links binocular rivalry switch rate to attention and subjective arousal levels in humans. Psychopharmacology, 195(3), 415-424.

Carhart-Harris, R. L., Kaelen, M., Whalley, M. G., Bolstridge, M., Feilding, A., & Nutt, D. J. (2015). LSD enhances suggestibility in healthy volunteers. *Psychopharmacology*, *232*(4), 785-794.

Carhart-Harris, R. L. (2018). The entropic brain-revisited. *Neuropharmacology*, *142*, 167-178.

Carter, O., Pettigrew, J., Hasler, F., Wallis, G., & Vollenweider, F. (2010). Psilocybin slows binocular rivalry switching through serotonin modulation. Journal of Vision, 6(6), 43.

Castro, A., Brenlla, M., & Casullo, M. (2002). Evaluación del bienestar psicológico en adultos argentinos. Evaluación del bienestar psicológico en Iberoamérica. Buenos Aires: Paidós.

Cohen, S., Kamarck, T., & Mermelstein, R. (1994). Perceived stress scale. Measuring stress: A guide for health and social scientists, 10(2), 1-2.

Del Cul, A., Baillet, S., & Dehaene, S. (2007). Brain dynamics underlying the nonlinear threshold for access to consciousness. PLoS biology, 5(10), e260.

Gough, H. G. (1979). A creative personality scale for the adjective check list. Journal of personality and social psychology, 37(8), 1398.

Guilford, J. P. (1967). The nature of human intelligence.

Haijen, E. C., Kaelen, M., Roseman, L., Timmermann, C., Kettner, H., Russ, S., ... & Carhart-Harris, R. L. (2018). Predicting responses to psychedelics: a prospective study. *Frontiers in pharmacology*, 897.

Jackson, S. A., & Marsh, H. W. (1996). Development and validation of a scale to measure optimal experience: The Flow State Scale. Journal of sport and exercise psychology, 18(1), 17-35.

Johnson, M. W., Richards, W. A., & Griffiths, R. R. (2008). Human hallucinogen research: guidelines for safety. Journal of psychopharmacology, 22(6), 603-620.

Kotov, R. I., Bellman, S. B., & Watson, D. B. (2004). Short Suggestibility Scale.

López-Pérez, B., Fernández-Pinto, I., & García, F. J. A. (2008). TECA: Test de empatía cognitiva y afectiva. Tea.

Mednick, S. A., & Mednick, M.T. (1959). Remote Associates Test, college and adult form.

Martin, M. M., & Rubin, R. B. (1995). A new measure of cognitive flexibility. Psychological reports, 76(2), 623-626.

Mrazek, M. D., Phillips, D. T., Franklin, M. S., Broadway, J. M., & Schooler, J. W. (2013). Young and restless: validation of the Mind-Wandering Questionnaire (MWQ) reveals disruptive impact of mind-wandering for youth. Frontiers in psychology, 4, 560.

Passie, T., Seifert, J., Schneider, U., & Emrich, H. M. (2002). The pharmacology of psilocybin. Addiction biology, 7(4), 357-364.

Reitan, R. M., & Wolfson, D. (1995). Category Test and Trail Making Test as measures of frontal lobe functions. The Clinical Neuropsychologist, 9(1), 50-56.

Shapiro, K. L., Raymond, J. E., & Arnell, K. M. (1997). The attentional blink. Trends in cognitive sciences, 1(8), 291-296.

Spielberger, C. D. (1983). State-trait anxiety inventory for adults.

Stroop, J. R. (1935). Studies of interference in serial verbal reactions. Journal of experimental psychology, 18(6), 643.

Tellegen Absorption Scale (TAS). A 34-item scale developed to measure the capacity of an individual to become absorbed in the performance of a task (Tellegen and Atkinson, 1974).

Watson, D., Clark, L. A., & Carey, G. (1988). Positive and negative affectivity and their relation to anxiety and depressive disorders. Journal of abnormal psychology, 97(3), 346.

Wallach, M. A., & Kogan, N. (1965). Modes of thinking in young children.
